# Q fever is an old and neglected zoonotic disease in Kenya: a systematic review

**DOI:** 10.1186/s12889-016-2929-9

**Published:** 2016-04-05

**Authors:** J. Njeru, K. Henning, M. W. Pletz, R. Heller, H. Neubauer

**Affiliations:** Institute of Bacterial Infections and Zoonosis, Friedrich-Loeffler-Institut, 07743 Jena, Germany; Center for Infectious Diseases and Infection Control, Jena University Hospital, 07740 Jena, Germany; Centre for Microbiology Research (CMR), Kenya Medical Research Institute, P. O. Box 19464-00202, Nairobi, Kenya; Center for Molecular Biomedicine, Institute for Molecular Cell Biology, Friedrich Schiller University Jena, 07745 Jena, Germany

**Keywords:** Seroprevalence, Epidemiology, *Coxiella burnetii*, Q fever, Kenya

## Abstract

**Background:**

Q fever is a neglected zoonosis caused by the bacterium *Coxiella burnetii*. The knowledge of the epidemiology of Q fever in Kenya is limited with no attention to control and prevention programs. The purpose of this review is to understand the situation of Q fever in human and animal populations in Kenya in the past 60 years, and help identify future research priorities for the country.

**Methods:**

Databases were searched for national and international scientific studies or reports on Q fever. We included studies and reports published between 1950 and 2015 if they reported on Q fever prevalence, incidence, and infection control programs in Kenya. Data were extracted with respect to studies on prevalence of *Coxiella* infections, study design, study region, the study populations involved, and sorted according to the year of the study.

**Results:**

We identified 15 studies and reports which qualified for data extraction. Human seroprevalence studies revealed evidence of *C. burnetii* infections ranging from 3 to 35.8 % in all regions in which surveys were made and two Q fever outbreak episodes. *Coxiella burnetii* infections found in cattle 7.4–51.1 %, sheep 6.7–20 %, camels 20–46 %, and goats 20–46 % revealed variation based on ecoregions and the year of study. Farming and lack of protective clothing were associated with increased seropositivity among humans. However, high quality data is lacking on Q fever awareness, underlying cultural-economic factors influencing *C. burnetii* infection, and how the pathogen cycles may be embedded in livestock production and management systems in the economically and ecologically different Kenyan regions. We found no studies on national disease incidence estimates or disease surveillance and control efforts.

**Conclusion:**

*Coxiella burnetii* infections are common in human and in a wide range of animal populations but are still unrecognized and underestimated thus presenting a significant human and animal health threat in Kenya. The factors influencing pathogen transmission, persistence and spread are poorly understood. Integrated disease surveillance and prevention/control programs are needed in Kenya.

## Background

Q fever is an acute (on occasion chronic) zoonotic disease of great public health importance worldwide. The disease is caused by an obligate gram-negative bacterium; *Coxiella burnetii* [1]. *Coxiella (C). burnetii* belongs to the genus *Coxiella* of the gamma subdivision of *Proteobaccteria* along with the genera *Legionella*, *Francisella*, and *Rickettsiella.* Unlike the other members of *Proteobaccteria, C. burnetii* is highly resistant to adverse physical conditions and chemical agents, so it can survive for months and even years in the environment. Its preferred target cells are macrophages located in body tissues and the monocytes circulating in the blood stream [2]. *C. burnetii* exists in two distinct antigenic forms, the phase I and phase II bacterial variants which can be discriminated by the surface lipopolysaccharide (LPS) composition. This antigenic variation is important for serological diagnosis and pathogenesis. Phase I variants are the highly infectious forms found in naturally infected hosts whereas phase II variants are less infectious and are obtained after serial passages in cell culture systems or embryonated eggs [3].

Domestic animals such as cattle, sheep and goats act as the major reservoirs of *C. burnetii* which can infect a large variety of animals, humans, birds, and arthropods [4–7]. Human infection results from inhalation of contaminated aerosols, consumption of contaminated unpasteurized dairy products, direct contact with contaminated milk, urine, feces, or semen of infected animals, and tick bites [8, 9]. Clinical presentation is nonspecific and highly variable ranging from asymptomatic infection (60 %) or self-limiting febrile illness associated with fatigue, headache, general malaise, myalgia, arthralgia, to atypical pneumonia(rapidly progressive courses may occur) and/or hepatitis. Less frequent complications include endocarditis, osteomyelitis and aseptic meningitis. About 1–2 % of acute symptomatic cases may develop chronic disease [10, 11]. Q fever is considered to be an occupational disease of people who have intimate contact with animals or their products such as veterinarians, farmers, abattoir workers, and laboratory workers [12]. There is emerging evidence of *C. burnetii* as a cause of non-malaria febrile illness and community acquired pneumonia in many developing countries [13–18], but hospital based diagnosis of Q fever in Kenya is uncommon. The lack of attention is mainly due to scarcity of available data and the perceived low clinical relevance of Q fever in relation to other endemic fevers. As a result, the disease might often be underreported and the disease burden grossly under-estimated [19].

In animals, Q fever is frequently asymptomatic. Sheep and goats may exhibit abortion, stillbirth, premature delivery, and delivery of weak offspring while cattle and camel may develop infertility, metritis, and mastitis [20, 21]. Animal studies have demonstrated that vaginal mucus, feces, and urine are the common shedding route and means for environmental contamination through kidding and effluent mismanagement [22, 23]. Mammals also considerably shed *C. burnetii* in milk and thus consumption of contaminated unpasteurized milk or dairy products can be a significant source of human infection [24]. Similar to humans, Q fever is under appreciated as cause of animal disease in Kenya possibly leading to persistence of the infection in animal herds, impacting on livestock productivity, and acting as sources of infection for humans [15].

The epidemiology of Q fever in Kenya is poorly understood due the apparent neglect of the disease by both medical and veterinary personnel and the limited capacity to enable meaningful epidemiological surveys. Therefore, we reviewed the literatures on Q fever among human and animal populations in Kenya, from 1950 to 2015 to understand the epidemiological features of the disease. The study also hoped to derive appropriate lessons from the Kenyan situation, and to identify the existing knowledge gaps on the *C. burnetii* infections in humans and animals, and the disease control programs.

## Methods

### Search

Databases including CABDIRECT, Science Direct, PubMed, and Google scholar were used for the searches. Available unpublished reports and theses from Kenyan university libraries and government departments were systematically searched. Other related articles emerging during the searches were also considered as sources of additional information. The searched publications were reviewed and relevant information was retrieved. The following keywords were used to perform the searches: ‘*Coxiella* in Kenya’ ‘Q fever in Kenya’ ‘Q fever in humans and animals in Kenya’, ‘*Coxiella* in humans and animals in Kenya’ and ‘zoonotic diseases in Kenya’. Studies or articles were included if they provided information on Q fever/*Coxiella burnetii* prevalence, disease incidence or outbreak, and infection control programmes in Kenya. Bibliographies of selected papers were also reviewed and grey literature searches performed for additional information. The studies or reports with unrelated reports were then excluded. The collected studies were reviewed and classified based on the study group (animals, humans and arthropods) and sorted according to the year of the study. All studies in humans indicating either acute or chronic disease were included. The seropositivity was used to describe serologic reactions that met the recommended titer cut-offs of the respective test method used. The clinical disease status was reported if the test method used meets the present guidelines for case definition [25].

## Results

### Data acquisition

**Fig. 1:**
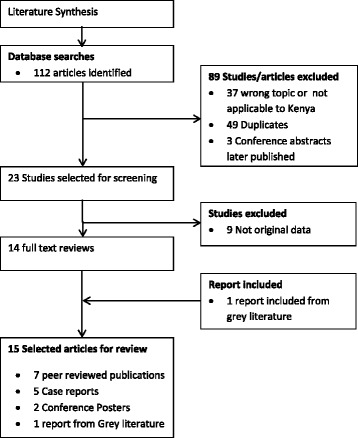
Search strategy and paper selection flowchart

In this review, 15 articles including published papers, conference abstracts/posters, student theses and government reports were ultimately identified and reviewed. These included nine (9) studies on humans [26–34], four (4) on animals [35–38], two (2) on ticks [17, 39], and two (2) studies on both animals and humans [17, 29] (Fig. 1). These studies differed in the methodological designs such as sample size, method of sample selection and collection, diagnostic criteria, and had limitations associated with the small scale cross-sectional epidemiological studies. For instance, five of these reports were case studies; two were outbreak reports whilst three studies were retrospective sero-epidemiological studies. The latter utilized either banked sera collected or submitted to research laboratories or specialized hospitals for disease diagnosis and did not provide proof of random sampling, hence sampling bias may have been significant in these studies.

### Evolution of the number of publications

**Fig. 2:**
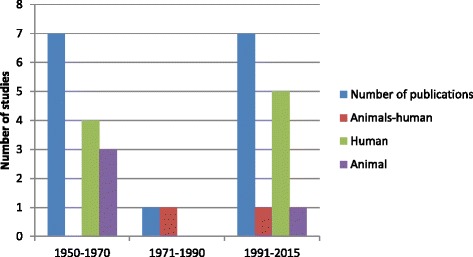
Number of publications and study groups included in the review, per 20 years period

The first publication of Q fever dates back to 1950s. The period from 1950 and 1976 was characterized by eight regional studies. This was then followed by a 32 year period (1971–2008) of no prevalence/incidence, except for an outbreak report documented in 2000. A slow increase of regional studies was found between 2008 and 2014 (Fig. 2).

### Disease attributed to *C. burnetii* in humans (Q fever)

The first human case of Q fever was reported in 1952 in a human clinical case in Nairobi [26]. Between 1955 and 1956, 17 more patients admitted to hospitals in Nairobi and Nakuru towns were found to have Q fever. These presented with febrile illness previously suspected and treated for viral pneumonia, malaria or other tropical fevers. Eventually, laboratory screening revealed that they were seropositive for *C. burnetii*. All these patients with exception of one were adult immigrants [27, 28]. A study conducted from 1967 to 1968 using archived sera obtained from hospitals in Western, Rift Valley, Central, Eastern, and Coast provinces of Kenya demonstrated an average seroprevalence of 35.8 % [29]. In the study, *C. burnetii* antibodies were detected in samples from all the participating hospitals. Two Q fever outbreaks were described in 2000 and 2014 involving 50 safari travelers [30] and 31 individuals in a small village in Rift Valley Province [31], respectively. Six deaths were reported in the latter outbreak. One of five Q fever cases diagnosed in 2008 among western travelers/tourists returning from tropics with fever was from Kenya [32]. A seroprevalence survey on banked sera of febrile patients treated in a rural clinic in western Kenya recorded seropositivity of 30.9, with 3 % of patients who had been diagnosed with acute lower respiratory infections (ALRI) being found to have acute Q fever [17]. A study by Cook et al. [33] reported *C. burnetii* seroprevalence of 4.5 % in abattoir workers in western Kenya while Mwololo et al. [34] found 26.8 % seropositivity in individuals in Bura district where mixed crop farming and pastoralism are practiced. The findings in these studies were recently presented at international conferences [33, 34].

### *Coxiella burnetii* infection in animals (Coxiellosis)

Serological surveys conducted in 1956 revealed 33.8, 7.4 and 75 % (3 of 4 dogs) seroprevalence in sheep, cattle and dogs, respectively from Central province, while North Rift region showed seroprevalence of 20 and 51.1 % in camels and cattle respectively [35, 38]. Brotherston and Cook (1956) found a higher *C. burnetii* seroprevalence (51.1 %) in cattle from the same region in the same year [28]. The study of Heisch et al. [36] found 2.6 % of cattle and 6.7 % sheep to be seropositive for *C. burnetii* in parts of Eastern province [36]. The study also showed a prevalence of about 13 % in 12 different species of wild rodents. The bacteria were detected in 81 organ pools (brain or spleen) of seropositive *Lemniscomys* spp. A 1976 study using banked sera from domestic animals (cattle, sheep and goats) from five administrative provinces (Western, Rift Valley, Central, Eastern, and Coast) showed that the prevalence in each of the regions ranged from 10–42.5 % [29]. Recently, Knobel et al. [17] reported high *Coxiella* seropositivity in cattle (28.3 %), goats (30 %), and sheep (18.2 %) in the western region [17]. A more recent study in mixed domestic animal-wildlife ranches of lower rift region found variation in seroprevalence among domestic animals; camels (46 %), goats (40 %) and sheep (20 %), respectively [37].

### *Coxiella* in ticks

Two studies described the presence of *Coxiella* in ticks. In 1962, Heisch et al. isolated *C. burnetii* by inoculating guinea-pigs with samples of ticks obtained from rodents in Nairobi, Rift Valley, and Eastern Provinces [39]. The study by Knobel et al. [17] detected *Coxiella* DNA in 50 % of tick pools obtained from domestic animals in Western Kenya. The highest prevalence was found in *Haemaphysalis leachi* [17].

### Risk factors

The study by Mwololo et al. (2014) on possible risk factors for Q fever infections in humans, identified increased age and farming as risk factors for seropositivity [34], while Cook et al. (2014) identified alcohol intoxication status of abattoir workers at work and lack of appropriate protective clothing as risk factors for *Coxiella* infection [33].

### Q fever diagnosis

Previous studies and reports in Kenya mainly utilized Complement Fixation Test (CFT) [26–29, 35–39] and only recently Enzyme-Linked Immunosorbent Assay (ELISA) [17, 31, 33, 34] and Immuno Fluorescence Assay (IFAT) [17] diagnostic assays were used to detect the presence of *Coxiella* antibodies in humans and animals. The study by Heisch et al. [39] demonstrated seroconversion of guinea pigs inoculated with ticks and organ pool samples obtained from wild rodents towards a range of *rickettsial* pathogens including *Coxiella* [39]. We found no study in which cell culture or PCR assays (that are widely considered as the gold standard for diagnosis for Q fever) were used.

### Q fever control programmes: One health approach

The government of Kenya in realization of the need for concerted inter sectoral (one health) approach in control of zoonotic diseases, formed the Kenya Zoonotic Diseases Unit (ZDU) in 2011. This intergovernmental unit aimed to establish collaborative programmes for effective prevention of zoonotic diseases in Kenya. However, we found no study or reports describing Q fever vaccination or disease awareness/control programmes in Kenya during our searches. At the time of this review, the disease was neither a notifiable disease nor listed in the priority zoonotic diseases list for Kenya [31].

## Discussion

The past surveys show strong evidence of Q fever exposure in animals and humans in all regions in which studies were conducted and the seroprevalence rates appear high. This is in contrast to the scarce literature available (Table 1). Though the first Q fever case was reported 63 years ago in Kenya [26, 35], only limited high quality epidemiologic studies utilizing randomly sampled human-animal populations for *Coxiella* have been conducted. Available studies were mainly retrospective using archived samples either submitted to reference laboratories or collected in hospitals for other diseases diagnosis resulting in sampling bias. We found no descriptions of official national disease surveillance and reporting or control programs in the published literatures. This knowledge gap highlights the need for more representative epidemiological studies in the country to elucidate the exact disease burden and pathogen transmission dynamics involved. The high seroprevalence of human antibodies against *Coxiella* antigens in the previous studies (3–35.8 %) suggests that cases are often misdiagnosed and/or unrecognized potentially leading to wrong patient management. This is a great challenge also in many sub-Saharan Africa countries where other tropical fevers such as malaria, typhoid, rotavirus and pneumonia are endemic. These infections present with similar symptoms making their diagnosis difficult leading to systematic under-reporting in the human health system [40, 41]. Studies in neighboring countries have reported comparable *Coxiella* seropositivity in humans, i.e., in Tanzania (3.9–20.3 %) [13, 19, 42], in Ethiopia (6.5 %) [43], and in Sudan (10 %) [44].

**Table 1:** Reviewed studies and reports of *C. burnetii* infection in humans and animals in Kenya

Study year	Region	Species	Prevalence/reactors	Inclusion criteria	Reference
1952	Central	Human	1 case	Case study	Harris, B.P
1955	Rift valley	Human	13 cases	Case study	Craddock, A.L. and J. Gear
1956	Central	Human	4 cases	Case study	Brotherston, J.C. and E.R. Cooke
	Rift valley	Cattle	20 of 35 cases		
1956	Central	Cattle	7.4 %	Retrospective epidemiological study	Brown, R.D
	Central	Goats	33.8 %		Vogel L.C et al.
	Rift valley	Camel	20 %		
	Central	Dogs	(3 of 4) cases	Case study	
1960	Multiple regions	Rodent	13 %	Seroepidemiological study	Heisch, R.B.
1962	Eastern	Cattle	2.6 %	Seroepidemiological study	Heisch, R.B., et al.
	Eastern	Sheep	6.7 %		
	Multiple regions	Ticks	2.3 %		
1976	Western	Human	45.7 %	Retrospective epidemiological study	Vanek, E. and B. Thimm
		Cattle	32.9 %		
		Goats	30		
	Rift valley	Human	20.3 %		
		Cattle	22.9 %		
	Central	Human	12 %		
		Cattle	10 %		
	Eastern	Human	41 %		
		Cattle	31.9 %		
	Coast	Human	50.4 %		
		Cattle	42.5 %		
2000	Rift valley	Human	8 %	Outbreak investigation	Potasman, I., et al.
2008	Unknown	Human	1 case	Case study	Ta, T., et al.
2013	Western	Human	3 %	Epidemiological study	Knobel, D.L., et al.
		Human	30.9 %	Retrospective epidemiological study	
		Cattle	28.3 %		
		Goats	30 %		
		Sheep	18.2 %		
		Ticks	50 %		
2014	Rift valley	Camel	46 %	Epidemiological study	DePuy, W., et al.
		Goats	40 %		
		Cattle	20 %		
2014	Coast	Human	26.8 %	Seroepidemiological study	Mwololo D. K et al.
2014	Western	Human	4.5 %	Seroepidemiological study	Cook, E.A. et al.
2014	Rift valley	Human	54.8 %	Outbreak investigation	ZDU, Kenya

Q fever is widely classified as an occupational disease for those who have close contact with animals or their products. Lack of appropriate personal protective clothing, present or past residing in a farm are reported to increase ones odds for *Coxiella* seropositivity [9, 12]. Farming, alcohol intoxication status of abattoir workers and lack of appropriate protective clothing during work were associated with seropositivity in Kenya [33, 34]. However, high quality data related to Q fever awareness or underlying social-economic or cultural factors predisposing humans to *C burnetii* infection are lacking. No studies were found on populations considered at high risk of infection such as veterinarians, retail butchers, pastoralist communities, and mixed livestock-crop production farmers. A study in Egypt on high risk groups reported high seropositivity (16 and 22 %) in populations living in close contact with animals and those living in rural establishment [45]. In contrast, a study in Chad found low seropositivity (1 %) in similar high risk groups [46]. A study in Tanzania demonstrated an association between Q fever onset and dry season [18]. Our review did not identify investigations on how the pathogen cycles are potentially embedded in livestock production and management systems in the economically and ecologically heterogeneous regions of Kenya.

**Fig. 3:**
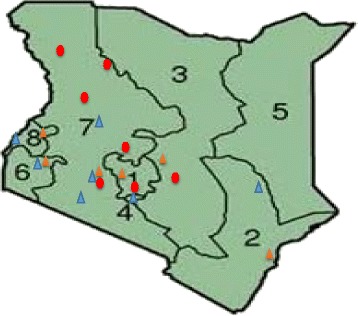
Map of Kenya showing eight administrative provinces (numbered) and spatial distribution of the previous studies. Key: Blue triangle: human studies; yellow triangle: linked human and animal studies; red dots: animal studies; 1: Central; 2: Coast; 3: Eastern; 4: Nairobi; 5: North eastern; 6: Nyanza; 7: Rift valley; 8: Western

We identified no study in human populations from northern eastern and upper eastern regions of Kenya but the available animal reports from similar populations in north rift regions revealed high exposure rates of 51.1 % and 20 % in cattle and camel, respectively (Fig. 3) [29, 35]. Epidemiological studies are needed in these high risk regions of Kenya because the communities in these regions practice mixed livestock ranching-and-wildlife conservancy systems and predominantly individual livestock-based pastoralism with high rates of livestock-wildlife interaction. Seroprevalence of *C. burnetii* of 7.4–51.1 % in cattle, 20–46 % in goats, 6.7–20 % in sheep, 20 and 46 % in camel and 13 % in rodents have been reported [18, 29, 35, 37, 38]. We found no distinct variation in *C. burnetii* infections among the animal species and the different administrative provinces. However, there were seemingly higher infections (28.2–57.1 %) reported in livestock from arid and semi-arid ecoregions of Rift Valley, Coast, Western and Eastern provinces when compared to Central and Nairobi. Though the precise reason for this variation is not clear, it is possible that specific local variation in prevalence exist based on the differences in animal husbandry practices, human and livestock density patterns and the differences in livestock infection in each of the ecoregions. Therefore, if no appropriate control strategies are implemented, the infected animals may continue to serve as reservoirs and source of infection to the uninfected animals and humans. Moreover, data on other common domestic animals in Kenya such as pigs, donkeys, and cats are not available. Therefore, the role of these domestic animals or ruminant wildlife animals as reservoirs and transmission of *Coxiella* need to be investigated. Surveys in the neighboring countries have reported *Coxiella* seropositivity with similar ranges in domestic animals, i.e., in Tanzania (13.3 % cattle, 13.6 % goats, 17.1 % sheep) [42], Ethiopia (31.6 % cattle, 54.2 % goats, 90 % camel) [47] and Sudan (24 % goats, 40.4 % cattle, 53 % goats, 62.5 % sheep [48, 49]. This epidemiologic status may be linked to the unregulated cross-border livestock movements through trade routes and nomadic movements between these countries in search of pasture and watering points that may allow the entry and spread of infected herds. These findings call for further research to elucidate the epidemiology of Q fever in linked animal and human populations in large representative surveys (within this african region) to enable assess the correlations of the disease incidence with agricultural production systems, geographical and climatic conditions and the animal husbandly practices.

Cell culture of *Coxiella* in a variety of culture media is considered as the gold standard for diagnosis for Q fever. However, the process is usually time consuming and difficult. Moreover, *Coxiella* is a highly hazardous pathogen whose isolation requires biosafety level 3 (BSL3) laboratory facilities because of its high infectivity potential [9]. As a result, this requires huge investments in terms of specialized skills and equipment. These are not affordable in many developing countries such as Kenya and thus previous epidemiological studies based on bacterial isolation have not been feasible. Recently, a cell-free laboratory culture medium whose composition mimics that of the host cells phagolysosome has been developed and it is undergoing evaluation and validation [2]. This discovery may therefore permit culture based epidemiological studies in resource limited settings such as Kenya in future. Detection of C. *burnetii* DNA in various samples by a range of Polymerase Chain Reaction (PCR) assays is progressively becoming available and is considered useful especially in the acute clinical stages of the illness when seroconversion is not sufficient to be detected by serological methods [50]. Similarly, the cost of the respective equipment and technical expertise makes it difficult for adoption for routine hospital based testing or to enable seroepidemiological research in many developing countries. Despite the availability of these diagnostic facilities in specialized and research laboratories in Kenya, Q fever diagnosis is still not considered during routine human and animals’ disease diagnosis systems. This can be attributed to the fact that these facilities are located in major towns which are not readily accessible to the regional health care facilities. Efforts to establish a regional laboratory in at least each county with facilities for performing reliable serological assay such as ELISA or serum based real-time qPCR are highly recommended. This approach will shorten the time needed to gain a sensitive diagnosis in patients presenting with fever of unknown origin and enable appropriate management of the patients.

As shown for other countries, controlling of the disease in animals contributes significantly to the decreased incidence in humans [51]. Vaccination of animals against Q fever could therefore present an effective control method. Until now, several vaccines have been developed consisting of inactivated whole phase 1 bacteria. However, many were shown to be nonprotective and presented several limitations such as short term protection and the inability to distinguish between vaccinated and naturally infected animals. Currently, prevention of Q fever in some European countries includes animal vaccination with the non-fully licensed inactivated phase I vaccine, Coxevac (CEVA-Santé Animale, France), after a focus of Q fever has been determined [9]. Several different vaccines have been developed for use in humans. These are mainly composed of a live attenuated strain or subunit vaccines [2, 52]. Despite demonstrating antigenicity and immunogenicity in many trials, humans develop severe reactions to the vaccinations making many of these vaccines effective to only individuals that have no previous exposures. This necessitates extensive screening before administration to having a direct impact on the final cost of the vaccine [53]. Presently, the considered effective vaccine is a formalin killed whole-cell vaccine (Qvax, CSL Limited, Australia) that is licensed for use in Australia, especially in high risk occupational groups. The availability of the vaccines present a great challenge in most of the developing countries and is currently not performed in Kenya.

### Limitations of the data

Lack of national level surveys and individual studies not being representative might lead to inappropriate estimates of Q fever prevalence. Also, the authors of the included studies have not taken into account the confounding bias and this may affect the true estimate.

## Conclusions

This is the first review of Q fever disease in animals and humans in Kenya. The findings from past literatures strongly suggest that *C. burnetii* infections are common in both humans and animals. Social-economic factors and lack of disease surveillance/control programmes present a significant risk of pathogen persistence and transmission in human and animal populations. More research is needed to elucidate the epidemiology of Q fever in linked animal and human populations in order to understand in greater details the human and animal exposure in different ecoregions and the correlations with disease incidence in different agricultural production systems. As with all zoonotic diseases, close collaboration between veterinary and medical authorities (One Health) both on national and regional levels is necessary in order to establish an integrated health surveillance and prevention/control programs for the disease in Kenya.

## Abbreviations

ALRI: acute lower respiratory infections
BSL3: biosafety level 3
CDC: centers for disease control and prevention
CFT: complement fixation test
ELISA: enzyme-linked immunosorbent assay
IFAT: immuno fluorescence assay
LPS: lipopolysaccharide
PCR: polymerase chain reaction
ZDU: zoonotic disease unit
